# Analysis of Auditory Function before and after a Single Session of Hemodialysis in Patients with Chronic Kidney Disease

**DOI:** 10.1055/s-0044-1789196

**Published:** 2025-05-12

**Authors:** Érica Alessandra Caldas, Patrick Rademaker Burke, Aline Gomes Bittencourt, Patricia Andréia Caldas, Eduardo Henrique Costa Rodrigues, Natalino Salgado Filho

**Affiliations:** 1 Programa de Ciências da Saúde, Universidade Federal do Maranhão (UFMA), São Luis, MA, Brazil; 2 Universidade Federal do Maranhão (UFMA), São Luís, MA, Brazil; 3 Department of Speech Therapy, Universidade Estadual Paulista (UNESP), Marília, SP, Brazil; 4 Department of Environmental Sciences, Universidade Estadual Paulista Júlio de Mesquita Filho (UNESP), São Paulo, SP, Brazil; 5 Universidade Ceuma (UNICEUMA), São Luís, MA, Brazil; 6 Hospital Universitário da Universidade Federal do Maranhão (HUUFMA), São Luís, MA, Brazil

**Keywords:** hemodialysis, hearing tests, hearing loss

## Abstract

**Introduction** Hearing is a complex process that involves mechanical, chemical, and neurophysiological components. Changes in hearing can be caused by congenital or acquired etiological factors. Chronic kidney disease (CKD) is one of the causes of hearing loss.

**Objective** To compare auditory findings before and after a single session of hemodialysis in patients with chronic kidney disease.

**Methods** A clinical cross-sectional research was conducted with a sample of 23 individuals between 24 to 57 years of age with a diagnosis of CKD undergoing hemodialysis. Distortion product otoacoustic emission (DPOAE) and transient otoacoustic emission (TOAE) tests were performed before and after a session of hemodialysis.

**Results** The DPOAE test revealed that 26% of the participants had failure in both ears prior to dialysis and 30.4% had failure after dialysis. Comparing the DPOAE and TOAE tests before and after hemodialysis, a slight decrease was found in patients with “fail” results from the predialysis test to the postdialysis test, but the difference did not achieve statistical significance.

**Conclusions** No significant hearing changes assessed through otoacoustic emissions occurred after a single session of hemodialysis in the sample analyzed.

## Introduction

Hearing is a complex process that involves mechanical, chemical, and neurophysiological components. Changes in hearing can be caused by congenital or acquired etiological factors, with various degrees and types of hearing loss. One possible cause is chronic kidney disease (CKD). Studies report that the prevalence of neurosensory hearing loss among patients with CKD ranges from 28 to 77%, 1 2 which is considerably higher than the rate found in the general population.

Although the association between CKD and hearing loss has been reported, the physiopathological mechanics of this condition have not been investigated. 3 4 5 The kidneys and cochlea are similar in regard to both structure and physiological mechanisms, involving the transport of fluids and electrolytes, which may explain hearing loss in individuals with CKD. 6 7

Other causes of hearing loss in this population have been considered, such as factors related to the severity and duration of CKD, electrolyte disorders, the use of ototoxic drugs, advanced age, comorbidities (diabetes mellitus and hypertension), and hemodialysis. 1 8 9

The systematic frequency of hemodialysis sessions can induce electrolytic, biochemical, immunological, osmotic, and vascular changes that can alter the functioning of the inner ear. 10 Therefore, the aim of the present study was to compare the auditory findings of patients diagnosed with CKD before and after a single session of hemodialysis.

## Methods

The present clinical cross-sectional study received approval from the Human Research Ethics Committee (approval number: 123.444) and was conducted in compliance with Resolution 466/2012 of the National Board of Health. All volunteers agreed to participate by signing a statement of informed consent.

Data collection took place between June 2022 and March 2023. Auditory findings were obtained before and after a single session of hemodialysis in patients with a diagnosis of CKD using distortion product otoacoustic emission (DPOAE) and transient otoacoustic emission (TOAE) tests with the aid of the Ero-Scan Pro (MAICO Diagnostics, Eden Prairie, MN, USA), duly calibrated following the ANSI S3.6 standard.

Twenty-three adults with CKD undergoing hemodialysis, irrespective of the time elapsed since diagnosis, were included. The exclusion criteria were age older than 60 years, individuals with altered hearing prior to the diagnosis of CKD, a history of exposure to noises of strong intensity, a family history of deafness, history of infection by rubella, cytomegalovirus, syphilis, herpes, hepatitis B, hepatitis C, or human immunodeficiency virus (HIV), individuals having undergone or currently undergoing treatment for cancer, and individuals having received a kidney transplant.

The otoacoustic emission (OAE) tests were performed by the same speech therapist in a silent room prior to and immediately after the end of the hemodialysis session. The following procedures were employed:

- Test in QuickScreen mode (rapid format with 12 milliseconds analysis window).
- Global analysis of responses for both TOAE and DPOAE, using the pass/fail protocol.
- Transient otoacoustic emission measured at frequencies of 1.5, 2.0, 2.5, 3.0, 3.5, and 4.0 KHz through 83 dB NPS click stimulus (duration ∼ 64 seconds). To be considered present, the signal/noise ratio (SNR) needed to be ≥ 6 dB at frequences between 1.0 and 4.0 KHz, and responses for at least 3 frequencies were required.
- Distortion product otoacoustic emission measured using 2 simultaneous pure tones with different frequencies at intensities P1 = 65 dB NPS and P2 = 55 dB NPS at frequencies of 2.0, 3.0, 4.0, and 5.0 KHz. Distortion product otoacoustic emission analyses were performed per frequency with the following criteria: amplitude (SD) greater than −5 dB and SNR higher than 6 dB.

The data were expressed as absolute and relative (%) frequencies. The Student *t* -test for paired samples was used to determine differences in the results before and after hemodialysis. The Chi-squared test was used to test possible associations with a 95% confidence interval (CI). The significance level was set at 5% ( *p* < 0.05). All analyses were performed with the aid of the SPSS Statistics for Windows, version 13.0 (SPSS Inc., Chicago, IL, USA).

## Results

A total of 30 patients were recruited, three of whom met one or more exclusion criteria and four who did not return for the follow-up exam after hemodialysis. Thus, the final sample comprised 23 individuals—12 women (52.1%) and 11 men (47.8%). Patient's ages ranged from 24 to 57 years, with a mean and standard deviation of 41 ± 9.82 years. The average duration of hemodialysis was 9.17 years (range: 1–36 years). With regards to comorbidities, 4 patients (17.3%) had diabetes mellitus and 17 (73.9%) had arterial hypertension. All patients had 3-weekly sessions of hemodialysis, with an average of four hours per session ( Table 1 ).

**Table 1:** Demographic and clinical characteristics of sample ( *n* = 23)

Variables	Female n (%)	Male n (%)	Total n (%)
**Demographic**			
Age group (years)			
24–30	3 (13.0)	1 (4.34)	4 (17.3)
31–40	4 (17.3)	3 (13.0)	7 (30.4)
41–50	5 (21.7)	3 (13.0)	8 (34.7)
51–57	0 (0.0)	4 (17.3)	4 (17.3)
**Clinical**			
Duration of dialysis (years)			
1–10	9 (39.1)	7 (30.4)	16 (69.5)
11–20	1 (4.34)	2 (8.6)	3 (13.0)
21–30	1 (4.34)	2 (8.6)	3 (13.0)
31–40	1 (4.34)	0 (0.0)	1 (4.34)
			
Diabetes			
Yes	3 (13.0)	1 (4.34)	4 (17.3)
No	9 (39.1)	10 (43.4)	19 (82.6)
Hypertension			
Yes	10 (43.4)	7 (30.4)	17
No	2 (8.6)	4 (17.3)	6
**Auditory symptoms**			
Tinnitus	3 (13.0)	4 (17.3)	7 (30.4)
Hearing loss	2 (8.6)	3 (13.0)	5 (21.7)
**Total**	12 (52.1)	11 (47.8)	23 (100)

Source: Author, 2023.

There were 7 patients (30.4%) with complaints of tinnitus, 6 of whom (26.0%) described the frequency of the symptom as sporadic, occurring mainly at the end of the hemodialysis session and characterized as mild. Also, 5 patients (21.7%) reported hearing loss, and 100% had no knowledge of hearing impairment being linked to kidney disease.

Furthermore, 6 patients (26.0%) failed in both ears during the predialysis DPOAE test, and 5 (21.7%) failed during the postdialysis test. There were 7 patients (30.4%) who failed during the predialysis TOAE test, and 7 (30.4%) failed during the postdialysis test ( Table 2 ). In the analysis of patients who “passed” or “failed” in both ears on the DPOAE and TOAE tests, no associations were found with age group or duration of hemodialysis.

**Table 2:** General responses on pre and postdialysis OAE tests using pass/fail system

	GENERAL OAE RESPONSES							
	DPOAE *n* = 23				TOAE *n* = 23			
Side	Pre-dialysis		Post-dialysis		Pre-dialysis		Post-dialysis	
	Pass n %	Fail n %	Pass n %	Fail n %	Pass n %	Fail n %	Pass n %	Fail n %
BEs	13 (56.5)	6 (26.0)	12 (52.1)	5 (21.7)	10 (43.4)	7 (30.4)	13 (56.5)	7 (30.4)
RE	14 (60.8)	9 (39.1)	16 (69.5)	7 (30.4)	13 (56.5)	10 (43.4)	16 (69.5)	7 (30.4)
LE	17 (73.9)	6 (26.0)	14 (60.8)	9 (39.1)	13 (56.5)	10 (43.4)	13 (56.5)	10 (43.4)

Abbreviations: BEs, both ears; DPOAE, distortion product otoacoustic emission; LE, left ear; RE, right ear; TOAE, transient otoacoustic emission; OAE, otoacoustic emission.

A greater number of failures were found for TOAE in both the right and left ears compared with DPOAE. Failures were predominantly in the left ear for DPOAE in the postdialysis test and in the left ear for TOAE in both the pre and postdialysis tests.

In the analysis of the frequencies tested, a greater number of patients failed at the highest frequencies (4.0 and 5.0 KHz) on both the pre and postdialysis tests ( Table 3 ). A discrete reduction was found in the number of patients who failed at all frequencies in the right ear between the pre and postdialysis tests. In contrast, an increase was found in the number of patients who failed at all frequencies in the left ear between the pre and postdialysis tests. However, the Student *t* -test revealed that the differences did not achieve statistical significance ( *p* > 0.05).

**Table 3:** Distribution of pre and postdialysis results (pass/fail) of DPOAE by ear ( *n* = 23)

Frequency (KHz)	Fail RE		Fail LE	
	Pre (%)	Post (%)	Pre (%)	Post (%)
2.0	5 (21.7)	4 (17.3)	1 (4.34)	5 (21.7)
3.0	6 (26.0)	4 (17.3)	7 (30.4)	8 (34.7)
4.0	10 (43.4)	9 (39.1)	9 (39.1)	10 (43.4)
5.0	10 (43.4)	9 (39.1)	6 (26.0)	7 (30.4)

Abbreviations: DPOAE, distortion product otoacoustic emission; LE, left ear; RE, right ear.

Note: Student *t* -test p > 0.05.

The results of the TOAE analysis for the number of patients who failed at each frequency are displayed in Table 4 . A greater number of patients failed at frequencies of 3.0, 3.5, and 4.0 KHz for both the right and left ears. In the comparison of the pre and postdialysis tests, an increase was found in the number of patients who failed at frequencies of 1.5 and 4.0 KHz for the right ear and frequencies of 2.5 and 3.5 KHz for the left ear. The results also show a reduction in the number of patients who failed on the postdialysis tests at frequencies of 2.0, 3.0, 3.5, and 4.0 KHz for the right ear and frequencies of 1.5, 2.0, and 4.0 KHz for the left ear. However, no statistically significant differences were found in the comparison of the pre and postdialysis tests ( *p* > 0.05).

**Table 4:** Distribution of pre and postdialysis results (pass/fail) of TOAE by ear ( *n* = 23)

Frequency (KHz)	Fail RE		Fail LE	
	Pre (%)	Post (%)	Pre (%)	Post (%)
1.5	8 (34.7)	10 (43.4)	11 (47.8)	7 (30.4)
2.0	10 (43.4)	8 (34.7)	10 (43.4)	8 (34.7)
2.5	11 (47.8)	11 (47.8)	12 (52.1)	14 (60.8)
3.0	16 (69.5)	13 (56.5)	15 (65.2)	15 (65.2)
3.5	15 (65.2)	13 (56.5)	16 (69.5)	19 (82.6)
4.0	19 (82.6)	12 (52.1)	12 (52.1)	10 (43.4)

Abbreviations: LE, left ear; RE, right ear; TOAE, transient otoacoustic emission.

Note: Student *t* -test p > 0.05.

An increase was found in the responses of the mean S/N ratio at all frequencies tested for the right ear and a frequency of 3.0 KHz for the left ear between the pre and postdialysis DPOAE tests ( Table 5 ).

**Table 5:** Mean signal/noise ratios of DPOAE ( *n* = 23)

S/N ratio	2.0		3.0		4.0		5.0	
RE	Pre	Post	Pre	Post	Pre	Post	Pre	Post
Mean	12.2	16	15.8	16	15.4	16.1	16.7	17.2
SD	10	8.7	8.7	8.2	9.5	9.0	11.1	9.3
Minimum	−10.3	−3	−2.9	−3	0	0.1	−6	−1
Maximum	30.2	30.5	31.7	29.3	32.2	31.2	32.9	31
LE								
Mean	15	13	16	16.2	17.3	15	18.8	17
SD	8.7	8.7	9.3	7.2	10	10.2	9.3	9.2
Minimum	2	0	−1	0	−4	−1	−1.1	−1
Maximum	34.2	30.6	30.1	26.3	30.2	29.6	27	28.3

Abbreviations: DPOAE, distortion product otoacoustic emission; LE, left ear; RE, right ear; SD, standard deviation.

Source: Author, 2023.

In the analysis of mean SNR for pre and postdialysis TOAE, an increase in values was found at frequencies of 2.5, 3.0, and 3.5 KHz for the right ear and frequencies of 1.5, 2.0, 2.5, and 3.0 KHz for the left ear ( Table 6 ).

**Table 6:** Mean signal/noise ratios of TOAE ( *n* = 12)

S/N ratio	1.5		2.0		2.5		3.0		3.5		4.0	
RE	Pre	Post	Pre	Post	Pre	Post	Pre	Post	Pre	Post	Pre	Post
Mean	12.4	5	9.6	5	−5.4	5	−1.4	4.6	1.1	4	4.4	3
SD	7.5	6.3	4.8	4.2	6.7	4.9	5.6	5.5	3.2	5.1	2.9	4.5
Minimum	−7	−3	−2	−2.1	−6	−3.3	−10	−48	−5	−8.1	−4	−3.8
Maximum	15.8	19.8	13.6	13.6	13	14.7	15	15	11.5	12	7	13.2
LE												
Mean	4.9	7	4	5	2.4	6	3.8	4	3.9	2	6	5.7
SD	4.9	4	4.6	4.6	5.8	4.8	3.8	4.5	4.3	3.9	4.9	4.9
Minimum	−3	−1	−3	−3	−6	−3	−12	−6	−5	3.5	−4.6	−3.5
Maximum	19.6	16.1	15.4	15.4	14.5	16.4	12.7	12.7	12.2	12.2	13	13

Abbreviations: LE, left ear; RE, right ear; SD, standard deviation; TOAE, transient otoacoustic emission.

Source: Author, 2023.

## Discussion

The hearing of patients with CKD has been widely studied in different countries in recent years, with important results demonstrating the risk of hearing loss in this population. 11 12 13 14 15 16 The most common finding is bilateral neurosensory hearing loss, with higher frequencies affected more and a notch at 6 KHz. 17

Similar results were found in the present study, as a larger number of patients failed at the frequencies of 4.0 and 5.0 KHz for DPOAE and frequencies of 3.0, 3.5, and 4.0 KHz for TOAE. The sample was composed of 12 women and 11 men, and no significant associations were found with sex, age group, or duration of hemodialysis.

The authors of a case-control study also identified neurosensory hearing loss in a sample of 50 patients with CKD and found no associations between hearing loss and disease duration or age. 14 However, Wu et al. 18 found that the risk of hearing loss increased considerably with the duration of the disease.

The type of treatment and its relationship to hearing loss is another point widely discussed in the literature. Patients on hemodialysis have better hearing results compared with those undergoing conservative treatment (medicinal therapy). 12

Although no statistically significant differences were found between the OETs conducted before and after hemodialysis, a discrete reduction was found in the number of patients who failed on the postdialysis tests. In 2001, Şerbetçioğlu et al. 19 studied 19 adults submitted to the high-frequency tonal audiometric test 1 hour before and 2 hours after hemodialysis and found no significant effect on hearing after a single session.

It is noteworthy that OAE tests have greater specificity for the assessment of cochlear function than other exams. These tests enable identifying cochlear disfunction prior to the change in audiometry. 20 21 22 Ozturan and Lam 23 also used OAE tests before and after a single session of hemodialysis and found no effect on hearing.

The reduction in the number of patients who failed the postdialysis test in the present investigation has been reported in previous studies. 24 Hemodialysis corrects a large part of hydroelectrical and metabolic changes of the endolymph induced by CKD and promotes the stabilization and correction of metabolic changes caused by the disease, leading to an increase in neural conduction and restoring the function of ciliated cells. 1 25 According to Gafter et al., 26 dialysis may be beneficial to hearing, but the effect is temporary.

Although numerous studies have associated hearing loss and CKD, few investigated the symptom of tinnitus. This symptom is defined as consciously perceptible sound in the absence of an external auditory stimulus. 27 Like hearing loss, tinnitus has a negative impact on quality of life. 28 In a retrospective cohort study involving 185,430 patients with CKD, Shih et al. 29 concluded that the disease constitutes a significant, independent risk factor for tinnitus and that patients with terminal kidney disease on dialysis are at greater risk of tinnitus than patients with CKD not undergoing dialysis.

In the present study, 30.4% of the patients reported tinnitus as an auditory symptom. The etiology of tinnitus can be considered multifactorial. Patients with arterial hypertension, diabetes mellitus, heart failure, and liver cirrhosis are at greater risk of tinnitus, and many of these factors are commonly found in individuals with CKD.

## Conclusions

No statistically significant auditory changes were found after a single session of hemodialysis in the sample analyzed. However, the number of patients who failed on the DPOAE and TOAE responses diminished following hemodialysis, characterizing an improvement in hearing.
